# Dietary saturated fat and low-grade inflammation modified by accelerometer-measured physical activity in adolescence: results from the GINIplus and LISA birth cohorts

**DOI:** 10.1186/s12889-019-7113-6

**Published:** 2019-06-25

**Authors:** Carla P. Harris, Andrea von Berg, Dietrich Berdel, Carl-Peter Bauer, Tamara Schikowski, Sibylle Koletzko, Joachim Heinrich, Holger Schulz, Marie Standl

**Affiliations:** 1 Institute of Epidemiology, Helmholtz Zentrum München, Institute of Epidemiology – German Research Centre for Environmental Health, Ingolstädter Landstr. 1, 85764 Neuherberg, Germany; 2 Dr. von Hauner Children’s Hospital, University Hospital, LMU of Munich, Munich, Germany; 3 Research Institute, Department of Pediatrics, Marien-Hospital Wesel, Wesel, Germany; 4 Department of Pediatrics, Technical University of Munich, Munich, Germany; 5 IUF-Leibniz Research Institute for Environmental Medicine (IUF), Düsseldorf, Germany; 6 Institute and Outpatient Clinic for Occupational, Social and Environmental Medicine, Inner City Clinic, University Hospital of Munich (LMU), Munich, Germany; 7 Allergy and Lung Health Unit, Melbourne School of Population and Global Health, The University of Melbourne, Melbourne, Australia; 8 Comprehensive Pneumology Center Munich (CPC-M), German Center for Lung Research, Munich, Germany

**Keywords:** Dietary intake, Low-grade inflammation, Saturated fat, Physical activity, Accelerometer, Adolescents, Epidemiology

## Abstract

**Background:**

Saturated fatty acids (SFA) have been reported to promote inflammation. Nevertheless, evidence linking dietary SFA and low-grade inflammation in adolescents is scarce and inconsistent. The modulatory role of physical activity (PA) on fat metabolism and inflammation may provide a potential explanation. Thus, we assessed the association of dietary SFA with high-sensitivity C-reactive protein (hsCRP), a marker of low-grade inflammation, in 15-year-olds, and evaluated possible interactions between dietary SFA and different levels of PA.

**Methods:**

Children participating in the 15-year follow-ups of the GINIplus and LISA German birth cohort studies were included (*N* = 824). SFA intake was estimated by means of a food frequency questionnaire and PA recorded by accelerometers. Average daily minutes of PA were classified into “sedentary”, “light” and “moderate-to-vigorous” (MVPA), using Freedson’s cut-offs. HsCRP concentrations were measured in serum and categorized into 3 sex-specific levels (below detection limit (I), above 75th percentile (III), in between (II)). Sex-stratified cross-sectional associations between SFA and hsCRP were assessed using multinomial logistic regression, adjusting for potential confounders. Interaction terms were included between SFA and the different PA levels; and if significant interactions were observed, analyses stratified by tertiles of the relevant PA levels were performed. Relative risk ratios (RRR) and 95% confidence intervals (95%CI) were presented for a 1% increase in SFA.

**Results:**

An inverse association was observed between SFA intake and hsCRP (II vs. I) in males (RRR = 0.85 [95%CI = 0.76;0.96], *p* = 0.008), whereas no significant association was observed in females. A significant interaction was observed with “sedentary” and “light” PA but not with MVPA in both sexes (*p* < 0.05). Stratified analyses indicated a significant inverse association between SFA and medium hsCRP levels in males in the highest light PA tertile (hsCRP II vs. I: 0.67 [0.517;0.858], *p* = 0.002).

**Conclusion:**

Our findings do not support a detrimental role of dietary SFA in low-grade inflammation among adolescents. In males, higher dietary SFA was associated with lower hsCRP, although this should be interpreted in the context of possibly correlated nutrients. Children spending the most time in light PA drove the observed inverse association, suggesting a synergistic effect of SFA and lifestyle PA in the resultant inflammatory response.

**Electronic supplementary material:**

The online version of this article (10.1186/s12889-019-7113-6) contains supplementary material, which is available to authorized users.

## Background

Chronic, low-grade inflammation precedes the onset of cardiovascular diseases [1], which continue to be among the leading causes of death and disability worldwide [2]. Ample evidence indicating that cardiovascular disease (CVD) risk can begin as early as childhood and track into adulthood [3, 4] has drawn research interests towards early biomarkers expressed in youth that may be predictive of adult morbidity [5]. The inflammatory marker high-sensitivity C-reactive protein (hsCRP) is used to predict the risk of atherosclerosis and CVD in adults [6] and has been observed in association with arterial alterations in children [7]. Raised hsCRP levels, even within the low ranges normally observed in children and adolescents [8], are associated with intermediate risk factors for CVD, including obesity [9, 10], insulin resistance [11, 12], and metabolic syndrome [13, 14]. Furthermore, childhood hsCRP levels have been shown to independently predict adult levels [15], as well as metabolic syndrome in adulthood [16]. HsCRP measured in adolescents can therefore offer a possible early indication of future CVD risk. Given that poor diet and lack of physical activity (PA) are amongst the primary lifestyle contributors to CVD [17], the specific roles of both these aspects in low-grade inflammation have become of eminent public health relevance. For decades, a key nutritional guideline has been to limit consumption of saturated fatty acids (SFA) [18, 19]. Yet the underlying evidence for this recommendation has been questioned in a number of studies [20–22], fuelling an ongoing debate [23–26]. Amidst this discussion, SFA have received much attention for their ability to promote inflammatory processes in vitro [27–29]. In large observational studies, direct associations between serum or plasma SFA status with markers of low-grade inflammation have been observed in both overweight and lean adults [30–32] as well as in children [33, 34]. Further, the upregulation of several genes relating to inflammatory pathways has been reported in a number of clinical trials in humans following the consumption of SFA, as summarised in a recent review [35]. Nevertheless, epidemiological studies observing longer-term dietary SFA in relation to inflammatory markers do not conclusively support a proinflammatory role of the nutrient [32, 36, 37]. Processes following dietary intake, including digestion, absorption, uptake into tissues, and metabolism, all affect the ensuing fatty acid profile [38], and could partially explain the conflicting findings relative to dietary SFA.

It has been proposed that the interaction with other lifestyle factors might be relevant in determining whether (or to what extent) dietary SFA contributes to chronic low-grade inflammation [39]. PA is frequently discussed for its role in promoting a long-term anti-inflammatory response [40], the mechanisms of which are not entirely understood [41]. Various studies in children and adolescents have not confirmed a direct association between PA and hsCRP [42–45], although the benefits on metabolic function and overall health are undeniable [46]. Studies reporting significant associations in adolescents suggest a protective role of PA. In a sample of adolescents aged 13–16 years, vigorous PA was shown to be protective of elevated CRP in boys but not in girls, independent of weight status [47]. Whereas in a study of adolescents from 10 European cities, objectively-measured vigorous PA was suggested to play an indirect beneficial role through improved cardiorespiratory fitness [48]. An indirect role through altered energy metabolism may also be plausible; for example, studies have shown that skeletal muscle activity can influence fat oxidation [49]. This effect can vary based on the intensity and duration of the activity, as well as by sex, as differences in substrate metabolism have been described, with females oxidising fat more readily than males during exercise [50]. Hence, a sex-specific modulatory role of habitual PA in the relationship between dietary SFA and chronic low-grade inflammation is plausible, perhaps through long-term physiological changes at the cellular level [51]. To our knowledge, no study has been carried out addressing the integrated role of SFA and PA in adolescent females and males. Therefore, this study aims to assess the association of dietary SFA with hsCRP in a large population of 15-year-olds, as well as the possible modulatory role of different levels and duration of objectively-measured PA.

## Methods

### Participants

The present study used data from the 15-year follow-up assessments of the GINIplus (German Infant Nutritional Intervention plus environmental and genetic influences on allergy development) and LISA (Influence of Life-style related factors on the development of the Immune System and Allergies in East and West Germany) birth cohort studies. Details on the cohorts’ recruitment and follow-up strategies have been described previously and can be found elsewhere [52, 53]. Briefly, healthy full-term new-borns were recruited from selected obstetric clinics in Germany. The GINIplus cohort (*n* = 5991) was recruited in Munich and Wesel between 1995 and 1998, and consists of two study arms: an observation arm and an intervention arm. New-borns with a family history of allergy were invited for the intervention arm, in which children were randomised to receive one of three hydrolysed formulas or cow’s milk. The aim was to compare the effect of the different formulae vs. cow’s milk on allergy development in a double-blind controlled trial. Participants with a negative family history of allergy, and those who declined to take part in the intervention trial, were included in the observation arm. The LISA cohort (*n* = 3094 - originally 3097 but three removed consent) is a true population-based cohort, recruited in Munich, Wesel, Leipzig and Bad Honnef, between 1997 and 1999. In both studies, information on selected exposures and health outcomes were obtained by means of questionnaires and medical examinations carried out at various follow-up assessments. Exposures and outcomes relevant to the present analyses are described in detail below. Both cohort studies have been approved by their local ethics committees (Bavarian Board of Physicians, University of Leipzig, Board of Physicians of North-Rhine-Westphalia) and written consent was obtained from all participants’ families.

### Dietary SFA

Habitual dietary intake was assessed at the 15-year follow-up using a self-administered food frequency questionnaire (FFQ). The applied FFQ was designed at the 10-year follow-up for the estimation of food and nutrient intake in school-aged children over the past year. A detailed description of the FFQ development and its validation can be found elsewhere [54]. Briefly, a list of commonly consumed foods contributing to total energy and especially fatty acid intake, was compiled from food intake data obtained by 3-day weighed dietary records of German children from the DONALD (Dortmund Nutritional and Anthropometric Longitudinally Designed) study [55]; portion sizes and frequency categories were included in the style of the EPIC (European Prospective Investigation into Cancer and Nutrition) FFQ [56]. A pilot study was conducted to evaluate the comprehensibility and applicability of the resulting FFQ, which was then validated against a 24-h dietary recall (at a food group level and at a nutrient level) [54]. The final version of the FFQ was used in the current study, and is available from the corresponding author upon reasonable request. The FFQ was delivered to participants by post and included detailed instructions for its completion. Participants were asked to report their estimated usual frequency (nine categories: ‘never’, ‘once a month’, ‘2-3 times a month’, ‘once a week’, ‘2-3 times a week’, ‘4-6 times a week’, ‘once a day’, ‘2-3 times a day’ and ‘four times a day or more’) and portion sizes (common household measures or coloured photographs of different portion sizes) of the intakes of the 80 listed food items over the past twelve months [57]. Additionally, several questions were included on preferred fat and energy contents, preparation methods, diets and food preferences, buying habits and dietary supplement use. Participants were asked to complete the FFQ themselves with the support of whoever cooked at home, if needed. The study technical assistant could be contacted if any further clarification was required. A quality control procedure was applied based on recommendations by Willett et al. [58] for data cleaning in nutritional epidemiology described thoroughly in an earlier publication [57]. Total daily energy and nutrient intakes were calculated (in kcal/day) based on the German Food Code and Nutrient Database (BLS) version II.3.1 [59]. The relative contribution of SFA to the overall diet was calculated and expressed as a percentage of total daily energy intake (%EI).

### Physical activity

PA was measured at age 15 years using triaxial accelerometers (ActiGraph GT3X, Pensacola, Florida), worn on the dominant hip for seven consecutive days. The accelerometer has been validated for use in adolescents [60], and it has been shown that measurements on opposing hips are not significantly different from each other [61, 62]. Participants for accelerometry were recruited from the study centers Munich and Wesel. This includes all of the GINIplus cohort and 64% of the LISA cohort taking part in the 15-year follow-up. The accelerometry protocol, data management and quality control have been described previously in detail [63, 64]. Briefly, participants were asked to keep an activity diary during the days the accelerometer was worn, where they recorded all their activities over the course of the day using a detailed schedule. This was done in order to control for non-wear time as well as the plausibility of the recorded accelerometer data. Since the goal was to capture a representative measure of usual daily PA, participants were asked to do the measurement during a “normal” week, i.e. no holidays, travelling, sickness. The activity diary information was also used to exclude recorded days which were not representative of typical routine, as described in Pfitzner et al. [64]. After passing quality control, at least 10 h of recorded time (or 7 if subjects were awake for less than 10 h) were necessary for a recorded day to be considered valid. Subjects were required to have at least 3 valid recorded weekdays and one valid weekend day. Measured accelerations were converted into activity counts and stored at 1 Hz (resampled from 30 Hz). Activity counts were then classified into one of four intensity levels (“sedentary”, “light”, “moderate”, and “vigorous” PA) on a minute-by-minute basis, estimated according to the uniaxial cut-offs published by Freedson et al. [65]. For the current analyses, three levels of PA were evaluated: “sedentary”, “light” (representing lifestyle PA), and “MVPA” (the sum of “moderate” and “vigorous” PA). Average minutes per day spent on the different PA levels were calculated for each individual by dividing total recorded minutes by the number of valid recorded days.

### Chronic low-grade inflammation

Serum concentrations of hsCRP were measured in samples collected during the 15-year follow-up medical examinations, using the Roche (Mannheim, Germany) Tina-quant CRP (latex) high-sensitive assay, according to manufacturer instructions. Measured hsCRP concentrations were highly skewed, with many observations below detection limit (hsCRP 0.016 mg/dl). Given this non-normal distribution, data categorisation was required for analyses. The variable was hence categorised into three levels separately for girls and boys, considering all children with available hsCRP measurements: all adolescents with hsCRP levels below the detection limit (hsCRP < 0.016 mg/dl) were grouped in the lowest category (I); amongst those remaining, sex-specific 75th percentiles were determined (0.085 mg/dl in girls; 0.092 mg/dl in boys), and those below the 75th percentile, i.e. girls with hsCRP < 0.085 and boys with hsCRP < 0.092 mg/dl, were assigned to the middle category (II); those above the 75th percentile, i.e. girls with hsCRP ⩾0.085 and boys with hsCRP ⩾0.092 mg/dl, were assigned to the upper category (III). While hsCRP values between 0.3 and 1 mg/dl are usually deemed indicative of elevated low-grade inflammation and of higher risk of CVD in adults [66], these levels are rarely met in children [8]. Unlike in adults, the cut-offs applied in the present study do not enable the identification of specific values on which to define high CVD risk. Rather, they allow the comparison between different hsCRP levels in young, healthy adolescents, amongst whom disease risk markers may manifest only in subclinical form. This is relevant given that hsCRP has been associated with early CVD risk factors like obesity [9, 10] and insulin resistance [11, 12] in children; also in the present study population, hsCRP was significantly positively associated with body mass index in both sexes (data not shown). This indicates that even young individuals with higher hsCRP levels with respect to their peers may be at greater CVD risk later in life, especially since hsCRP levels track into adulthood [15]. General consensus suggests that hsCRP > 1 mg/dl likely reflects acute infection or trauma [66]. Values > 1 mg/dl were therefore not considered for the present analyses in order to avoid assessing acute inflammation.

### Statistical analyses

Participants from the 15-year follow-up of GINIplus and LISA studies, with complete data on SFA intake, accelerometer-measured physical activity, and hsCRP, were included in the statistical analyses. Since PA was measured only in Munich and Wesel, the study sample was limited to participants from these two study centres. Sex-stratified associations between SFA intake and hsCRP were assessed using multinomial logistic regression, adjusting for potential confounders: study (GINI intervention arm; GINI observation arm; LISA), region (Munich; Wesel), parental education (based on highest level achieved - low: ≤10th grade; high: >10th grade), pubertal stage (based on a self-rating Pubertal Development Scale (PDS), which has been validated in adolescents and includes ratings on body hair growth, voice change and facial hair growth for boys, and body hair growth, breast development and menarche for girls [67, 68]. For each item (except menarche which had a yes/no response) there were four response options ranging from “not yet started” to “seems complete”, which were used to create pubertal stage categories: early-; mid-; late-; post-pubertal), fasted blood sampling (yes; no), exact age at blood sampling (years), body mass index (BMI, in kg/m^2^ calculated from height and weight measurements obtained during physical examination, unless unavailable (*n* = 65), in which case obtained from the 15-year follow-up questionnaire), and total daily energy intake (kcal/day).

In three additional models, separate adjustment for the different PA levels (“sedentary”, “light” and “MVPA”) was carried out. Interaction terms were then included between SFA and the different PA levels. Where a significant interaction was observed, additional analyses were performed, stratified by tertiles of the relevant PA level. Here, we corrected for multiple testing using Bonferroni correction: the α-level was divided by three (the number of subgroups assessed for each sex in stratified analyses). This yielded a corrected two-sided α-level of 0.017 (0.05/3 = 0.017). Relative risk ratios (RRR) and 95% confidence intervals (95%CI) are presented for a 1% increase in SFA. The RRR indicates the risk of being in one of the comparison groups relative to the risk of being in the reference group given a 1% change in SFA. All analyses were conducted using R, version 3.3.2 (https://www.R-project.org/) [69]. Multinomial logistic regression was calculated using the “multinom” function in the R package “nnet” [70].

## Results

### Study population

A total of 824 participants (469 females, 355 males) were included in the analyses (see Additional file 1: Figure S1). Complete data on SFA intake, accelerometer-measured PA, and hsCRP, was available for 1011 children. Participants were excluded who were lacking data for adjustment variables (171 subjects), as well as those who reported an illness affecting diet or inflammatory status (8 subjects, 3 with diabetes, 2 with coeliac disease, 2 with cancer, 1 with Crohn’s disease). Children were also excluded if they presented clear outliers in SFA intakes (none) or in PA levels (2 subjects, 1 female with MVPA = 257 min/day, 1 male with MVPA = 220 min/day), as identified by visual inspection of descriptive plots. Finally, children with hsCRP values > 1 mg/dl (likely reflecting acute inflammation) were further excluded (6 subjects with hsCRP levels ranging from 1.12 mg/dl to 3.86 mg/dl). Basic characteristics of the study population are displayed in Table 1. Girls and boys presented no significant differences in hsCRP levels (mg/dl) or in SFA intakes (%EI). In contrast, girls spent significantly more time in “sedentary” activities than boys, whereas boys spent more time in “light” PA and “MVPA”, and consumed higher amounts of daily calories.

**Table 1:** Descriptive characteristics of the study population

	Females (*n* = 469)	Males (*n* = 355)	*P*-value
hsCRP [mg/dl]	0.04 (0.02; 0.07)	0.03 (0.02; 0.07)	0.438
*hsCRP catergories*			
I	64 (13.6)	59 (16.6)	0.492
II	312 (66.5)	227 (63.9)	
III	93 (19.8)	69 (19.4)	
SFA [%EI]	12.8 (3.0)	13 (2.9)	0.265
Sedentary [min/day]	602 (561; 645)	585 (531; 631)	**<0.01**
Light PA [min/day]	241 (209; 276)	258 (224; 291)	**<0.01**
MVPA [min/day]	34 (24; 46)	43 (30; 57)	**<0.01**
*Study*			
GINIplus intervention	173 (36.9)	112 (31.5)	0.057
GINIplus observation	182 (38.8)	131 (36.9)	
LISA	114 (24.3)	112 (31.5)	
Region [Munich]	279 (59.5)	251 (70.7)	**0.001**
Parental education [High]	340 (72.5)	265 (74.6)	0.540
*Pubertal stage*			
Early	0 (0)	21 (5.9)	**<0.01**
Mid	20 (4.3)	133 (37.5)	
Late	378 (80.6)	199 (56.1)	
Post	71 (15.1)	2 (0.6)	
Fasting Blood [Yes]	199 (42.4)	179 (50.4)	**0.027**
Age [years]	15.2 (0.3)	15.2 (0.3)	0.870
BMI [kg/m2]	20.3 (18.7; 22.1)	19.9 (18.5; 22.1)	0.141
Daily calories [kcal]	1846 (563)	2406 (680)	**<0.01**

Values are mean (sd) or median (25th percentile; 75th percentile) for continuous variables with normal and non-normal distribution, respectively, and n (%) for categorical variables. (I) hsCRP < 0.016 mg/dl; (II) hsCRP ⩾0.016 mg/dl and < 75th sex-specific percentile of those with hsCRP ⩾0.016 mg/dl (< 0.085 mg/dl in girls; < 0.092 mg/dl in boys); and (III) hsCRP ⩾75th sex-specific percentile of those with hsCRP ⩾0.016 mg/dl (⩾0.085 mg/dl in girls; ⩾0.092 mg/dl in boys). Differences between females and males were tested by Student’s t-test (means) or Wilcoxon’s rank-sum test (medians) for continuous variables, and by Pearson’s Χ^2^-test for categorical variables. Significant *p*-values are marked in bold (*p* < 0.05)

### Dietary SFA and hsCRP

Results from the multinomial logistic regression assessing the association between SFA and hsCRP, including individual adjustment for different PA levels, “sedentary”, “light” and “MVPA”, and their interactions, are displayed in Table 2. No statistically significant association was observed between SFA and hsCRP in females, whereas males presented an inverse association (hsCRP II vs I: RRR = 0.873 (95%CI = 0.78; 0.98), *p* = 0.021), which remained significant following further adjustment for PA variables. A dose-response relationship was however not indicated, as this association was observed at the middle hsCRP (II) level but not the upper (III) level.

**Table 2:** Associations between SFA and hsCRP categories adjusting for different PA levels (Sedentary, Light, MVPA)

	hsCRP II vs I				hsCRP III vs I			
	RRR	95%CI	p-value	p-int	RRR	95%CI	p-value	p-int
Females								
SFA^a^	0.970	0.88;1.07	0.543		0.966	0.861;1.09	0.563	
+Sedentary^b^	0.970	0.88;1.07	0.543	**<0.01**	0.967	0.861;1.09	0.565	**<0.01**
+Light^c^	0.969	0.88;1.07	0.538	**0.007**	0.965	0.859;1.08	0.546	**<0.01**
+MVPA^d^	0.970	0.88;1.07	0.546	0.519	0.968	0.862;1.09	0.577	0.658
Males								
SFA^a^	0.873	0.78;0.98	**0.021**		0.922	0.81;1.06	0.241	
+Sedentary^b^	0.879	0.78;0.99	**0.027**	**<0.01**	0.927	0.81;1.06	0.273	0.753
+Light^c^	0.873	0.78;0.98	**0.021**	**0.026**	0.922	0.81;1.06	0.241	0.627
+MVPA^d^	0.881	0.78;0.99	**0.034**	0.615	0.927	0.81;1.06	0.280	0.413

^a^Models adjusted for study, region, parental education, pubertal stage, fasted blood sampling, exact age at blood sampling, BMI, total daily energy intake; ^b^ SFA model further adjusted for Sedentary PA; ^c^ SFA model further adjusted for Light PA; ^d^ SFA model further adjusted for MVPA. Significant associations are marked in bold (*p*-value <0.05). p-int = *p*-value for interaction term between SFA and each PA variable, indicated as significant (bold) when <0.05

### Effect modification by PA

**Fig. 1:**
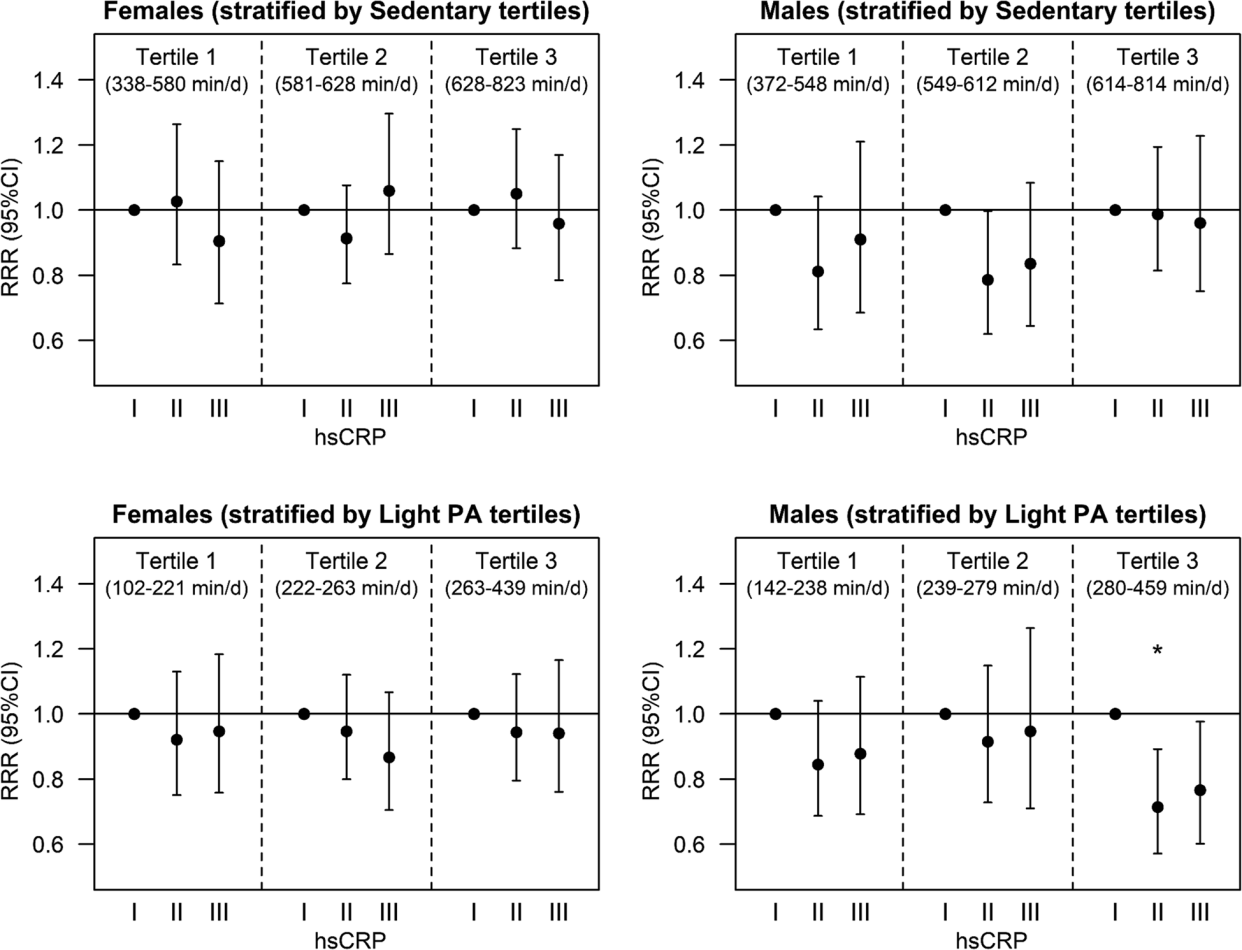
Associations between SFA and hsCRP stratified by tertiles of time spent in Sedentary activity (top plots) and in Light PA (bottom plots) in females and males (left and right, respectively)

While adjustment for PA levels did not influence the association between SFA and hsCRP, significant interactions with time spent “sedentary” and in “light” PA were observed in females and males. Further analyses were hence carried out stratified by both these PA levels. Results from the stratified analyses are displayed in Fig. 1 (exact values can be found in Additional file 2: Table S1). Analyses stratified by tertiles of “sedentary” time indicated no association between SFA and hsCRP in either females or males in any of the tertiles. On the other hand, analyses stratified by tertiles of time spent in “light” PA indicated an inverse association between SFA and hsCRP only for male subjects in the highest “light” PA tertile (hsCRP II vs I: 0.714 (0.57;0.89), *p* = 0.003).

## Discussion

The present study assessed the association between dietary SFA and low-grade inflammation, measured by the inflammatory marker hsCRP, in 15-year-old German adolescents. Two aspects stand out among the analyses findings: 1) dietary SFA appears to have no association with hsCRP levels in adolescent females and to be inversely associated with hsCRP in males, albeit only for the middle hsCRP level with respect to the lowest, thus showing no clear exposure-response relationship; 2) a significant interaction between SFA intake and “light” PA likely plays a relevant role, given that the inverse association observed in males was only present among those spending the most time in “light” PA, as indicated by stratified analyses. Although average daily minutes of MVPA were comparable to reports in other adolescent populations [71, 72], it should be kept in mind that the majority of adolescents in this study do not meet the WHO recommendation of 60 min of MVPA per day [73]. This is not uncommon for adolescents; according to the WHO, over 80% of 15-year-olds in European populations do not reach PA recommendations [74]. The present findings are therefore relevant to underscore the positive role of “light” PA in modifying potential inflammatory effects of diet in adolescents, who clearly struggle to reach adequate levels of MVPA. Nevertheless, different results might be expected in more active individuals, especially regarding interactions between MVPA and SFA, as levels may be too low in this population to detect significant effects.

### Dietary SFA and hsCRP

The results of our analyses in females are in line with a number of studies in adults, in which no significant association has been observed between dietary SFA and hsCRP [32, 36, 75]. Others have reported positive associations, supporting arguments to limit SFA intake in order to reduce cardiovascular risk [76–78]. Existing studies addressing children and adolescents are equally inconclusive. In a sample of 79 Swiss children aged 6–14 years, total dietary fat, but not one specific type of fat (mono- or poly-unsaturated fatty acids or SFA), was directly associated with subclinical inflammation [79]. Another study in a random sample of 12–17-year-old girls in the Balearic Islands (*n* = 219), reported no association between dietary SFA and hsCRP [80]. On the other hand, a population-based study including 602 children aged 5–13 years in a Brazilian city, showed a direct association between dietary SFA and high hsCRP, defined as CRP levels > 0.1 mg/dl [81]; while a study in 359 urban Asian Indian adolescents and young adults (87% males) observed twice the odds of having raised CRP levels (> 0.3 mg/dl) in subjects with intakes of SFA > 10%EI [82]. The inconsistency among the various study findings might be related to study location, which could influence habitual dietary behaviours and patterns, sources of dietary SFA, or even baseline hsCRP levels. With respect to this last point, the median hsCRP levels in our study population were 0.04 mg/dl in females and 0.03 mg/dl in males. Although no hsCRP reference values are yet available for adolescents, these levels are comparable to pre-pubertal reference values in Europe [8]. Intakes of SFA in our study population (around 13%EI) were also similar to intakes reported in other German adolescent populations [83]. Compared to the study in Asian Indians, who presented higher average CRP values (0.13 mg/dl), our results and those of the Spanish study might suggest that dietary SFA is not significantly relevant in females, within the ranges of hsCRP and SFA here reported. Nevertheless, this was not supported by results from the Brazilian study, which applied a much lower cut-off to define upper hsCRP levels (> 0.1 mg/dl) and still observed a direct association with SFA.

It is also possible that contradicting results in terms of SFA may reflect true differences between SFA *intake* and *status*. While the assessment of SFA *intake* considers the amount of the nutrient consumed, SFA *status* likely reflects circulating SFA following additional processes such as digestion, absorption, uptake into tissues, and metabolism [38]. In the present analyses, we observed an inverse association between SFA intake and middle hsCRP levels in males. Such an association between dietary SFA and hsCRP was also observed by Fredrikson et al. in adult females [84], a finding which the authors described as surprising. It has been proposed that the sparing of SFA and endogenous de novo SFA synthesis both contribute to SFA status and are promoted by high-carbohydrate diets [85]. Reduced SFA intakes have been shown to be compensated by a concomitant increase in carbohydrate (CHO) intakes [86]. Indeed, our dietary data presented strong negative correlations between SFA and CHO (r = − 0.76 and r = − 0.80 in females and males, respectively). Due to the strong correlations, adjustment for CHO in our statistical models was not possible as this would have led to problems of multicollinearity. We hence emphasize that these results should be interpreted in the context of other, possibly correlated nutrients. High glycaemic index CHO has been reported to induce inflammation through postprandial hyperglycaemia even in lean, glucose-tolerant subjects [87], and several intervention studies seem to support this notion [88]. A previous study including a subset of the present study population showed that the highest contribution towards total energy intake at age 15 years came from “refined grains” and “sugar-sweetened foods” [57]. We therefore speculate that increasing SFA intake likely has no direct role in reducing low-grade inflammation per se, but might promote a reduced inflammatory profile indirectly through a simultaneous reduction in CHO intake. This finding would hence support statements advocating that it is not simply the dietary SFA content, but the entire dietary composition, and especially the relative CHO intake, that determines whether SFA intake is ultimately associated with detrimental outcomes [89].

### Effect modification by PA

The inverse association between SFA and the middle category of hsCRP, apparent in male adolescents of the current study, was driven mainly by a specific subgroup with high levels of daily “light” PA. To our knowledge, this is the first study to evaluate the interaction of PA in the association of SFA and low-grade inflammation in adolescents, and hence comparison with other studies is limited. Anti-inflammatory effects of habitual PA in children have been observed [90], although a study assessing accelerometer-measured PA in 9-year-old children reported no association between PA and hsCRP [44]. None of the different PA levels assessed were significant confounders in our analyses, and therefore did not alter the inverse relationship between SFA and middle category levels of hsCRP when included in the statistical models as covariates. It could be suggested from the current findings that PA exerts part of its anti-inflammatory effects through its modifying role in SFA metabolism, with “light” PA being most relevant in this context. For a 1% increase in energy intake from SFA in males in the highest tertile of “light” PA, the relative risk of being in the middle hsCRP category vs. the lowest was reduced by a factor of 0.714. It is possible that MVPA was too low in our study population to induce significant synergistic effects with diet. In previous analyses of PA in the present birth cohorts, only about 1% of the subjects achieved the WHO recommended 60 min of MVPA per day [63]. On the other hand, it is known that the intensity of PA is the main factor determining the degree of CHO or fat oxidation for fuel, and that low-intensity exercise depends almost entirely on fatty acids [91]. This might explain why only “light” PA presented a significant interaction with SFA in our analyses. It seems plausible, that an increased capacity to metabolize lipids throughout the day (> 4.6 h of “light” PA were undergone on average daily in males in the highest tertile) could reduce the levels of circulating SFA, thereby limiting a possible pro-inflammatory response to higher SFA intakes compared to less active subjects. The inverse association was not significant in children with the highest hsCRP levels, although a near-significant trend was present (*p* < 0.05). It is possible that due to the smaller sample size and greater variance in the highest hsCRP category, there was insufficient power to detect a significant association. Why the inverse association was only observed in males is unclear, but it is possible that sex-specific physiological factors might play a significant role, leading to differences in fat metabolism and the resulting inflammatory profile. For example, testosterone has been shown to enhance lipid oxidation whereas oestrogen enhances fat storage [92], aspects which may be relevant in the context of the present study, especially considering that most of the females in the sample were in late- or post-pubertal stages. On the other hand, males were significantly more active than females and it is possible that females were not sufficiently active for a significant anti-inflammatory response with higher SFA intakes in synergy with “light” PA.

### Strengths and limitations

The present study benefits from a large, homogeneous study sample, and adds to the limited literature on the association between dietary SFA and low-grade inflammation in adolescents, in a time of heightened discussion concerning SFA and cardiovascular health. Our study includes data from over 800 individuals, greatly exceeding the size of the few observational studies carried out thus far. Our analyses also include the assessment of different levels of accelerometer-measured physical activity, a method not often available in large cohort studies. Accelerometers were worn by participants on the hip, reported to be the best single location to record data for activity detection [93]. Furthermore, to our knowledge, the role of SFA with regards to inflammation has not been previously assessed in the context of different PA levels and their possible interactions. The current analyses hint towards potential synergistic effects of important modifiable lifestyle factors in relation to health aspects, particularly in males. Their interaction may differ substantially from their individual effects and this can be highly relevant when interpreting findings on a topic such as SFA, on which contradicting results are often discussed. This study focusses on a population of healthy adolescents aged 15-years, which is not a high-risk population. Given the low levels of hsCRP being addressed, results are not necessarily indicative of damage nor directly translatable to CVD risk, and hence the clinical relevance of the present findings may seem limited. However, given the increasing evidence for the progression of risk factors from childhood to adulthood, preventive measures might already consider this age group and hence associations observed could provide valuable insight.

A main limitation when assessing dietary intake is the reliance on subjective measures, which are prone to reporting bias. In the present study the FFQ used to measure dietary SFA was designed to estimate fatty acids and antioxidants in school-aged children [54]. Given the thorough quality control of the dietary data (with plausible values observed in terms of total energy intake), misreporting was likely detected and excluded from the analysis. A further drawback is the high inter-correlation amongst different nutrients, which is often come across in nutritional epidemiology, and if ignored could lead to inappropriate conclusions. Adjustment for these nutrients within the statistical models could result in multicollinearity, generating further misleading associations [94]. With this in mind, and considering that the inclusion of an interaction term with PA would further complicate interpretation, we could not adjust for other nutrients and hence the ability to disentangle the individual effects of SFA is somewhat limited. Nonetheless, we are aware of the importance of accounting for possible intercorrelations and have hence considered these in the interpretation of our results. A further limitation in the present study was the underrepresentation of children from lower social-classes. As often occurs in longitudinal cohort studies, this non-random loss-to-follow-up meant that the current findings may not be entirely representative of the study area. The assessment of other inflammatory markers might have been useful to strengthen our conclusions, but unfortunately these were not available for the studied cohorts. Our findings are based on cross-sectional analyses, meaning that the observed associations between dietary SFA and hsCRP do not necessarily infer causality. Furthermore, blood-withdrawal for CRP measurements was carried out at a slightly different time to dietary assessment and accelerometry. Thus, the present analysis is based on the assumption that dietary intake, as well as PA and CRP, are persistent during this interval, which may not be entirely the case. Nevertheless, for PA, activity measured on non-typical days (e.g. including trips or sickness) were excluded to ensure usual activity was recorded, which more likely represents chronic PA. In terms of dietary intake, it was not possible to check if intakes of SFA directly prior to hsCRP measurement were indeed the same as those recorded in the dietary assessment; however, our focus was on chronic SFA intake and the FFQ was designed to estimate intakes over the past 12 months, which includes the time of blood withdrawal for most participants. We assume that any drastic changes in diet between FFQ completion and blood withdrawal are unlikely, although it cannot be entirely excluded. Nevertheless, changes occurring in either lifestyle behaviour would have occurred at the individual level, and hence any bias due to such changes are expected to be random, not affecting the general trend observed. Furthermore, the analyses were adjusted for age, which partially accounts for season, as participants were invited to follow-up assessments 2–4 weeks before their birth month, with the aim of limiting systematic bias.

## Conclusion

From the present analyses, it can be concluded that a higher SFA intake during adolescence, within the ranges observed in the current study, is not detrimental in terms of inflammatory processes in adolescents; although we highlight that this may well depend on the nutrient it replaces. Furthermore, the inflammatory role of SFA might be modulated by the amount of daily “light” PA. Adolescent males with higher SFA intakes, who also participated in greater amounts of “light” PA, presented lower levels of hsCRP than their less active peers. We propose that when evaluating the role of SFA in chronic inflammation, it is essential to differentiate between findings involving SFA status (in serum or plasma) and those assessing dietary SFA, as the latter is likely influenced by important modifiable factors such as PA, which may determine whether an inflammatory response arises.

## Additional files

Additional file 1: **Figure S1.** Study Participants. (PDF 188 kb)

Additional file 2: **Table S1.** Associations between SFA and hsCRP categories stratified by tertiles of PA (Sedentary and Light). (PDF 189 kb)

## Data Availability

The datasets generated and/or analysed during the current study are not publicly available due to data protection reasons, but are available from the corresponding author on reasonable request, provided it is consistent with the consent given by the study participants. In some cases, ethical approval can be obtained for the release. Lastly, a data transfer agreement must be accepted and the request must be approved by the studies’ steering committees. Requests should be addressed to Marie Standl (marie.standl@helmholtz-muenchen.de).

## Abbreviations

MVPA: Moderate-to-vigorous physical activity
PA: Physical activity
SFA: Saturated fatty acids
