# Supplementary figures and images for: Dietary saturated fat and low-grade inflammation modified by accelerometer-measured physical activity in adolescence: results from the GINIplus and LISA birth cohorts
Source: BMC Public Health. 2019 Jun 25;19:818. doi: 10.1186/s12889-019-7113-6 (PMC6593603; doi:10.1186/s12889-019-7113-6)

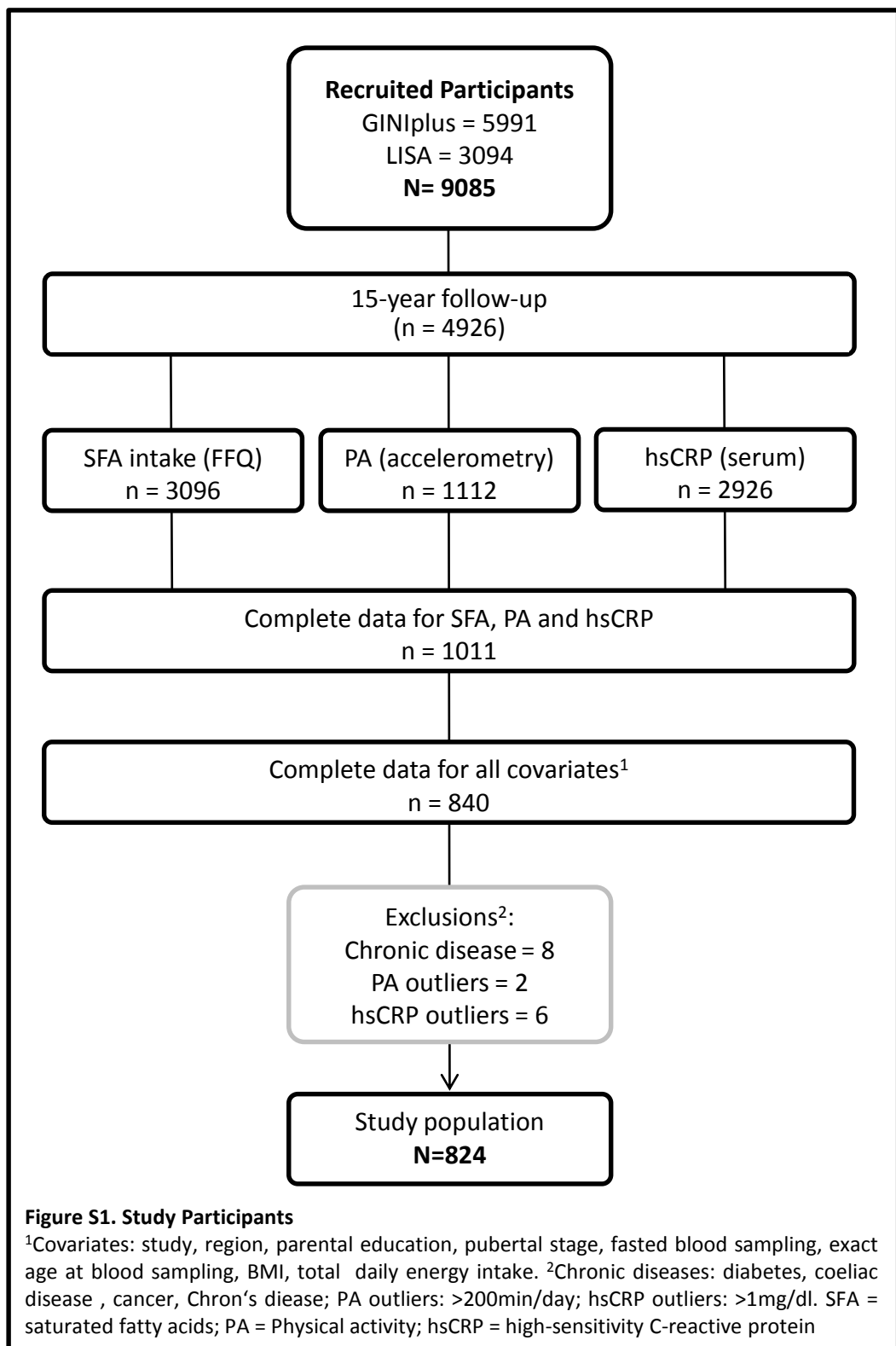

Supplement: Supplementary file 1 — Figure S1. Study Participants. (PDF 188 kb) [file 12889_2019_7113_MOESM1_ESM.pdf]
