# Supplementary material for: Dietary saturated fat and low-grade inflammation modified by accelerometer-measured physical activity in adolescence: results from the GINIplus and LISA birth cohorts
Source: BMC Public Health. 2019 Jun 25;19:818. doi: 10.1186/s12889-019-7113-6 (PMC6593603; doi:10.1186/s12889-019-7113-6)
Supplement: Supplementary file 2 — Table S1. Associations between SFA and hsCRP categories stratified by tertiles of PA (Sedentary and Light). (PDF 189 kb) [file 12889_2019_7113_MOESM2_ESM.pdf]

Table S1. Associations between SFA and hsCRP categories stratified by tertiles of PA (Sedentary and Light)

	**Females[a]**						**Males[a]**					
	hsCRP II vs I			hsCRP III vs I			hsCRP II vs I			hsCRP III vs I		
	RRR	95% CI	p-value	RRR	95% CI	p-value	RRR	95% CI	p-value	RRR	95% CI	p-value
**Sedentary**												
Tertile 1	1.026	0.83;1.26	0.809	0.905	0.71;1.15	0.415	0.812	0.63;1.04	0.100	0.910	0.69;1.21	0.517
Tertile 2	0.913	0.78;1.08	0.279	1.059	0.87;1.30	0.575	0.786	0.62;1.00	0.047	0.836	0.64;1.08	0.177
Tertile 3	1.050	0.88;1.25	0.583	0.958	0.79;1.17	0.672	0.987	0.82;1.19	0.890	0.960	0.75;1.23	0.747
**Light**												
Tertile 1	0.921	0.75;1.13	0.431	0.947	0.76;1.18	0.632	0.845	0.69;1.04	0.111	0.878	0.69;1.11	0.283
Tertile 2	0.947	0.80;1.12	0.524	0.867	0.71;1.07	0.176	0.915	0.73;1.15	0.442	0.947	0.71;1.26	0.713
Tertile 3	0.944	0.80;1.12	0.516	0.941	0.76;1.17	0.579	0.714	0.57;0.89	**0.003**	0.766	0.60;0.98	0.032

[a]Models adjusted for study, region, parental education, pubertal stage, fasted blood sampling, exact age at blood sampling, BMI, total daily energy intake.
Significant associations are marked in bold (Bonferroni-corrected p-value <0.017)
